# Implementation of a PEWS-integrated electronic SBAR handover model for pediatric inpatients: A nonconcurrent controlled study

**DOI:** 10.1097/MD.0000000000049883

**Published:** 2026-08-07

**Authors:** Huali Huang, Bo Liu, Xiaowan Gu, Zaoqiong Gao, Jiangbo Ba, Qiang Fu, Ting Chen, Shengnan Zhao

**Affiliations:** a Department of Pediatrics, Jingzhou Hospital Affiliated to Yangtze University, Jingzhou, Hubei, People’s Republic of China; b Gansu Provincial Maternity and Child-Care Hospital (Gansu Provincial Central Hospital), Lanzhou, Gansu Province, People’s Republic of China.

**Keywords:** electronic handover, nonconcurrent controlled study, patient safety, pediatric early warning score, pediatric nursing, SBAR

## Abstract

This study aimed to evaluate observed changes in pediatric inpatient handover quality, efficiency, satisfaction, and safety-related indicators after implementation of a pediatric early warning score-integrated electronic Situation-Background-Assessment-Recommendation (SBAR) handover model. This single-center nonconcurrent controlled study was conducted in 3 general pediatric wards of a tertiary hospital in Jingzhou, China. The control period was January to March 2024 with routine SBAR handover, and the intervention period was June to August 2024 after development, electronic integration, and staff training. The same 47 pediatric nurses participated in both periods. The control period included 206 children and 384 handover observations; the intervention period included 212 children and 395 observations. Outcomes included bedside handover quality, nurse handover evaluation, family bedside handover perception, handover errors, handover efficiency, unplanned pediatric intensive care unit (PICU) transfer, and nursing adverse events. Because of the nonconcurrent design, findings were interpreted as observed between-period differences rather than direct causal effects. During the intervention period, bedside handover quality, nurse handover evaluation, and family bedside handover perception scores were higher than during the control period (93.66 ± 9.52 vs 83.93 ± 8.86; 58.21 ± 7.24 vs 52.51 ± 5.21; 63.23 ± 3.51 vs 55.59 ± 3.71; all *P* < .001). The handover error rate was lower (6.84% vs 17.97%, *P* < .01). Report-writing time and bedside handover time were shorter (2.11 ± 0.41 vs 4.51 ± 0.32 minutes and 3.94 ± 0.63 vs 5.97 ± 0.31 minutes; both *P* < .001). The unplanned PICU transfer rate was numerically lower (0.94% vs 3.88%), but this finding was borderline and not statistically significant by Fisher’s exact test (*P* = .059). Nursing adverse events were less frequent during the intervention period (4.72% vs 11.17%, *P* = .015). Implementation of a pediatric early warning score-integrated electronic SBAR handover model was associated with better handover quality, higher nurse and family evaluations, improved efficiency, fewer handover errors, and fewer observed nursing adverse events. The lower unplanned PICU transfer rate should be interpreted cautiously. Multicenter prospective studies are needed to confirm these findings.

## 1. Introduction

Situation-Background-Assessment-Recommendation (SBAR) is a structured communication framework designed to improve the completeness, clarity, and consistency of clinical information transfer. It has been widely adopted in healthcare handover and escalation contexts because it encourages clinicians to communicate the current situation, relevant background, clinical assessment, and recommended actions in a concise sequence.^[1–4]^ In pediatric wards, where disease progression can be rapid and symptom presentation may be atypical, structured communication is particularly important for maintaining continuity of care.

Pediatric Early Warning Score (PEWS) systems provide a quantitative approach to recognizing early physiological deterioration in children. The Brighton PEWS and related pediatric early warning tools have been used to support timely risk recognition, escalation, and decision-making.^[5–7]^ Integrating PEWS into bedside handover may help nurses communicate not only descriptive information, but also risk-stratified assessment of a child’s current clinical status.

Electronic handover tools can reduce repetitive documentation and improve the standardization of information transfer by extracting selected clinical information from hospital information systems (HIS). However, many electronic handover forms remain based mainly on routine items and local experience rather than on a structured communication framework and validated early warning assessment. Studies combining SBAR, early warning scoring, handover templates, and information technology remain limited, especially in pediatric inpatient settings.^[8–12]^

This study implemented a PEWS-integrated electronic SBAR handover model in a pediatric department. The purpose was to compare handover quality, handover errors, nurse and family satisfaction, handover efficiency, unplanned pediatric intensive care unit (PICU) transfers, and nursing adverse events between a routine SBAR control period and a PEWS-integrated electronic SBAR intervention period. Because the study used a nonconcurrent controlled design rather than random allocation, the results were interpreted as observed between-period differences associated with implementation of the model.

## 2. Methods

### 2.1. Study design and setting

This was a single-center nonconcurrent controlled study conducted in 3 general pediatric wards of Jingzhou Hospital Affiliated to Yangtze University, a tertiary hospital in Jingzhou, China. The control period was January to March 2024, during which routine SBAR bedside handover was used. The intervention was developed, electronically integrated, and tested from April to May 2024. The intervention period was June to August 2024, during which the PEWS-integrated electronic SBAR handover model was implemented. The study was reported according to principles for observational studies.

### 2.2. Participants

The nursing participants were 47 pediatric nurses who worked in the 3 general pediatric wards throughout the study. There was no change in the nursing staff during the control and intervention periods. The nurses had a mean age of 33.60 ± 6.04 years and a mean work experience of 11.68 ± 6.80 years; all were female and all had a bachelor’s degree. Nurses were eligible if they were employed pediatric nurses, voluntarily participated, and had worked for at least 1 year. Nurses were excluded if they were advanced trainees or interns, or if they were on maternity leave, sick leave, or personal leave for at least 1 month during the study period.

Parents or legal guardians of hospitalized children were eligible if the child was newly admitted or critically ill with a hospital stay of at least 24 hours, if the caregiver could communicate normally and complete the questionnaire, and if informed consent was obtained. Children were excluded if they or their guardians requested withdrawal, if the child was under active resuscitation at the time of assessment, or if the child died during the study. The control period included 206 children and 384 handover observations; the intervention period included 212 children and 395 handover observations. Baseline characteristics of caregivers and children were compared between the 2 periods and showed no statistically significant differences in the variables available in the original dataset (Table 1).

**Table 1:** Baseline characteristics of caregivers and children in the control and observation groups.

Characteristic	Control group	Observation group	*χ*^2^/*t*	*P*-value
**Caregiver characteristics**				
Primary caregiver			1.471	.479
Parents	154 (74.80)	167 (78.80)		
Grandparents	36 (17.50)	28 (13.20)		
Others	16 (7.80)	17 (8.00)		
Caregiver education level			2.999	.392
Primary school or below	10 (4.90)	7 (3.30)		
Junior high school	35 (17.00)	28 (13.20)		
Senior high school	36 (17.50)	48 (22.60)		
College or above	125 (60.70)	129 (60.80)		
**Child characteristics**				
Sex			3.415	.065
Male	98 (47.60)	120 (56.60)		
Female	108 (52.40)	92 (43.60)		
Age, mo	36.58 ± 39.90	40.30 ± 38.57	−0.969	.333
Patient type			0.013	.910
New	84 (41.60)	87 (41.00)		
Severe	118 (58.40)	125 (59.00)		
Nursing level			3.185	.074
Level I nursing	119 (57.80)	104 (49.10)		
Level II nursing	87 (42.20)	108 (50.90)		
Disease category			7.054	.217
Respiratory diseases	137 (66.50)	127 (59.90)		
Systemic/infectious diseases	22 (10.70)	18 (8.50)		
Digestive diseases	21 (10.20)	28 (13.20)		
Nervous system diseases	10 (4.90)	10 (4.70)		
Blood/immune diseases	11 (5.30)	14 (6.60)		
Other diseases	5 (2.40)	15 (7.10)		

Data are n (%) or mean ± standard deviation. For patient type, valid percentages were retained as reported in the original table. The observation group nursing evel count for Level I nursing was corrected to 104 to match the reported percentage and *χ*^2^ statistic. All baseline comparisons were nonsignificant.

### 2.3. Ethical considerations

This study was approved by the Ethics Committee of Jingzhou Hospital Affiliated to Yangtze University (approval number: 2025-011-01). Informed consent was obtained from participating nurses and from the parents or legal guardians of the hospitalized children. The study used de-identified data for analysis, and all procedures complied with institutional requirements for clinical nursing research.

### 2.4. Sample size

For the nursing participants, cluster sampling was used to include all 47 eligible pediatric nurses in the participating wards. For hospitalized children, the sample size was calculated using handover report information accuracy as the primary reference indicator. Based on previous literature reporting an accuracy rate of 89.66% in the control group and 97.67% in the intervention group,^[12]^ with a 1:1 group ratio, α = 0.05, and β = 0.10, PASS 15.0 indicated that at least 158 cases were required in each group. After allowing for a 10% rate of loss or refusal, at least 174 cases were required per group. The final sample sizes of 206 children in the control period and 212 children in the intervention period exceeded this requirement.

### 2.5. Routine SBAR handover during the control period

During the control period, night-shift nurses used the routine SBAR handover form and recorded the patient’s condition in the handover book following the S-B-A-R sequence. During morning handover, the night-shift nurse-reported the ward’s general situation, critically ill patients, and newly admitted patients. The on-duty physician provided supplementary clinical information, and the head nurse and department director gave department-level instructions when needed. Medical and nursing staff then separated for bedside handover, during which each patient was handed over according to the routine SBAR process.

### 2.6. PEWS-integrated electronic SBAR handover model during the intervention period

A working group was established in November 2023 to develop the pediatric PEWS-integrated electronic SBAR model. The group included nursing quality management personnel, the pediatric department director, information technology personnel, the head nurse, and senior clinical staff. Based on literature review, expert discussion, and the characteristics of pediatric inpatient care, the group developed a modified pediatric SBAR handover template incorporating the Brighton PEWS.

The S (Situation) section included bed number, name, sex, age, diagnosis, diet, nursing level, main symptoms, vital signs, sleep, diet, elimination, psychological status, and current treatment. The B (Background) section included allergy history, medical history, and positive laboratory or examination findings. The A (Assessment) section included PEWS, skin condition, catheter status, pain, pressure injury risk, fall risk, nutrition, intravenous leakage or extravasation risk, potential complications, existing nursing problems, and current nursing interventions. The R (Recommendation) section included priorities for the next-shift, early identification and management of potential risks, and key nursing points. The structure of the PEWS-integrated SBAR pediatric handover template and its electronic or manual information sources are summarized in Table 2.

**Table 2:** PEWS-integrated SBAR pediatric handover template and electronic information sources.

SBAR/domain	Template content/key elements	Score or assessment weight/source category	Electronic or manual data source
Situation: general information	Bed number, name, diagnosis, diet, nursing level	General information, 15 points	System database timestamp; HIS physician order processing system
Situation: main condition	Vital signs, chief complaint, symptoms, signs, excretion, sleep, psychology	Main condition, 15 points	HIS medical record
Situation: diagnosis and treatment	Main treatment and oral medication	Diagnosis and treatment, 10 points	HIS physician order execution form
Background: positive indicators	Past medical history, allergy history, examination results, test results	Positive indicators, 15 points	HIS medical record; critical value records; imported abnormal tests; self-recorded items
Assessment: specialized condition	PEWS score, skin, pipes/catheters, risk score, nursing problems, potential complications	Specialized condition assessment, 20 points	Free entry; nursing record sheet; nursing plan form; manual revision
Assessment: accident risk	Burns, aspiration, wandering and other pediatric accident risks	Accident risk assessment, 15 points	Free entry; nursing record sheet
Disease evaluation	Pain, tenderness, diet, nutrition, venous evaluation/extravasation risk and other high-risk items	High-risk items displayed when applicable	Disease, tenderness, diet, nutrition, and venous evaluation scales
Recommendation	Observation points, nursing key points, next-shift priorities	Nursing suggestions and key points, 10 points	Free entry; manual corrections

HIS = hospital information system, PEWS = pediatric early warning score, SBAR = situation, background, assessment, recommendation.

The electronic handover report was developed by combining automatic HIS data extraction, manual entry, and nurse verification. Before handover, the outgoing nurse reviewed the automatically generated information, completed missing items, corrected any inaccurate content, printed the electronic handover report, and signed it. At bedside, the outgoing nurse greeted the child and caregiver and reported information in the S-B-A-R sequence. The incoming nurse confirmed and signed the report. If the child’s actual condition differed from the electronic report, the outgoing nurse provided immediate clarification and recorded the correction in the remarks section. Hand disinfection was performed after each bedside handover.

From April to May 2024, nurses received training on SBAR principles, pediatric PEWS scoring, electronic handover operation, risk escalation procedures, and standardized bedside communication. Training methods included lectures, case analysis, scenario simulation, and video demonstration. Nurses were required to pass a scenario-based bedside handover assessment before participating in the intervention period handover process.

### 2.7. Design considerations and potential confounding

Because this study used a nonconcurrent rather than randomized concurrent design, several potential sources of bias were considered. First, the control and intervention periods occurred in different seasons, and seasonal variation in pediatric disease patterns, ward workload, and patient acuity may have influenced outcomes. Second, the same nurses participated in both periods; therefore, learning and training effects may have contributed to improved performance during the intervention period. Third, the intervention was a multicomponent package that included a structured SBAR template, PEWS assessment, electronic information extraction, workflow standardization, and staff training. The study was designed to evaluate the implementation package as a whole and could not estimate the independent effect of each component. Fourth, the department reviewed internal practice records and did not identify other major ward-level handover or safety quality-improvement projects implemented during the study interval; however, unmeasured concurrent practice changes could not be fully excluded. These issues were considered when interpreting the findings, particularly for low-frequency outcomes such as unplanned PICU transfers and nursing adverse events. The study also considered the statistical instability of low-frequency safety outcomes; therefore, unplanned PICU transfer was interpreted with particular caution and was checked using Fisher’s exact test in addition to the originally reported *χ*^2^ test.

### 2.8. Outcome measures

Bedside handover quality was measured using the Nurse Bedside Handover Evaluation Scale, which includes 5 domains and 13 items with a total score of 100; a score of 85 or above indicates qualified handover quality.^[12]^ Nurse perception of handover was measured using the Chinese version of the Handover Evaluation Scale, which has reported Cronbach’s α coefficients of 0.956, 0.770, and 0.739 for its factors and a test-retest reliability coefficient of 0.910.^[13]^ Family perception of bedside handover was measured using the Chinese version of the Bedside Handover Perception Scale, which includes 3 dimensions and 17 items and has acceptable reliability and validity.^[14–16]^

Handover errors and omissions were assessed using a Nurse SBAR Handover Quality Checklist developed by the research team according to the PEWS-integrated SBAR template. The checklist included 20 core handover items covering identity information, vital signs, medication, nursing measures, PEWS and other risk assessments, abnormal findings, special test results, and family communication points. “Error” was defined as handover information inconsistent with the patient’s actual condition or medical record; “omission” was defined as failure to mention information that should have been handed over according to the patient’s condition and nursing standards. One quality-control nurse and one ward nurse manager independently performed random checks twice weekly after standardized training. Ten percent of handover observations (n = 78) were independently scored by both assessors; the kappa value was 0.86, indicating high inter-rater agreement.

Report-writing time was recorded by the night-shift nurse during the control period and was generated by the system during the intervention period after nurse verification and finalization. Bedside handover time was defined as the time from the start to the completion of bedside handover for each patient. Nursing adverse events included falls, aspiration or suffocation, burns, pressure ulcers, medication errors, tube dislodgement, infusion leakage, skin injury, and family complaints.^[17]^ Unplanned PICU transfer referred to transfer to PICU due to clinical deterioration and excluded routine planned PICU admission.^[18]^

### 2.9. Statistical analysis

Data were double-entered in Excel and analyzed using SPSS version 21.0. Continuous variables were assessed for normality using the Shapiro–Wilk test. Normally distributed variables were summarized as mean ± standard deviation; nonnormally distributed variables were summarized as median and interquartile range. Categorical variables were summarized as frequency and percentage. Baseline characteristics of children and caregivers were compared between the control and intervention periods using independent-samples *t* tests or Mann-Whitney *U* tests for continuous variables and *χ*^2^ tests or Fisher exact tests for categorical variables, as appropriate.

Nurse-reported handover quality and handover evaluation scores were collected from the same nursing workforce in the 2 periods and were analyzed as before-after period comparisons. Family-reported perception scores were compared between independent caregiver samples from the 2 periods. Handover observation-level indicators, including handover errors and time indicators, were analyzed according to the number of observed handover instances. Child-level safety indicators, including unplanned PICU transfer and nursing adverse events, were analyzed according to the number of hospitalized children in each period. For low-frequency categorical outcomes, Fisher’s exact test was additionally considered to avoid overinterpretation of *χ*^2^ results when expected counts were small. Two-sided *P*-values < .05 were considered statistically significant. Because of the nonrandomized nonconcurrent design, all analyses were interpreted as observed between-period differences rather than proof of direct causal effects.

## 3. Results

### 3.1. Baseline characteristics

A total of 47 pediatric nurses participated throughout both periods. The control period included 206 hospitalized children and 384 handover observations, and the intervention period included 212 hospitalized children and 395 handover observations. Baseline caregiver characteristics, including primary caregiver type and caregiver education level, were comparable between periods (both *P* > .05). Baseline child characteristics, including sex, age, patient type, nursing level, and disease category, were also comparable between periods (all *P* > .05; Table 1).

### 3.2. Bedside handover quality and nurse handover evaluation

The total bedside handover quality score was higher during the intervention period than during the control period (93.66 ± 9.52 vs 83.93 ± 8.86, *t* = −5.126, *P* < .001; Table 3). Most item scores were higher during the intervention period, including greeting and addressing the child and family, introduction of the handover team, main condition handover, vital signs/ECG monitoring, infusion status, skin condition, oxygenation, nebulization and suction, special tests or treatments, patient psychological status, and humanistic care/privacy-related handover behavior. The item for indwelling needle and tube fixation/patency was numerically higher during the intervention period but did not reach statistical significance (*P* = .054).

**Table 3:** Nurse bedside handover quality and nurse handover evaluation scores before and after implementation.

Scale/item	Control group (n = 47)	Observation group (n = 47)	*t*	*P*-value
**Nurse bedside handover evaluation scale**				
Greeting: properly addressing and informing the patient and family about handover	4.15 ± 0.72	4.79 ± 0.46	−5.103	<.001
Introduction: introducing oneself and the handover team	3.85 ± 1.06	4.43 ± 1.08	−2.601	.011
Main content: bed number, name, age, diagnosis, chief complaint, condition changes, treatment and medication, current condition, and night-shift sleep status	7.94 ± 1.54	9.02 ± 1.44	−3.534	.010
ECG monitoring: vital signs, consciousness, and pupil response	8.53 ± 1.25	9.56 ± 0.88	−4.584	<.001
Infusion: medication, amount, nature, drip rate, tube connections, infusion site, and infusion bag signature	4.34 ± 0.60	4.79 ± 0.51	−3.897	<.001
Indwelling needle and tube fixation and patency	8.87 ± 1.10	9.36 ± 1.33	−1.951	.054
Skin condition and device-related pressure/adhesive injury	8.79 ± 1.28	9.49 ± 1.04	−2.936	.004
Oxygenation improvement	8.70 ± 1.16	9.64 ± 0.74	−4.675	<.001
Nebulization and suction status	8.34 ± 1.29	9.38 ± 1.03	−4.324	<.001
Special tests or treatments during handover	8.81 ± 1.33	9.66 ± 0.73	−3.847	<.001
Patient psychological status	7.32 ± 1.78	8.98 ± 1.51	−4.869	<.001
Hand hygiene, position, familiarity with patient condition, humanistic care, and privacy protection	4.30 ± 0.62	4.57 ± 0.72	−2.001	.048
Total score	83.93 ± 8.86	93.66 ± 9.52	−5.126	<.001
**Nurse handover evaluation scale**				
I can receive the latest information	3.85 ± 0.72	4.36 ± 0.71	−3.470	.001
I can fully obtain information about the patient	3.83 ± 0.82	4.57 ± 0.54	−5.212	<.001
I can organize the information I receive clearly	4.23 ± 0.67	4.40 ± 0.65	−1.255	.213
The information provided by colleagues is easy to hand over	4.15 ± 0.69	4.43 ± 0.72	−1.908	.060
I always receive important information	4.00 ± 0.83	4.60 ± 0.61	−3.940	<.001
I can focus my attention on the information I receive	4.15 ± 0.72	4.45 ± 0.69	−2.020	.043
When I face difficulties during handover, I can consult colleagues	4.34 ± 0.73	4.70 ± 0.51	−2.788	.006
During handover, I have the opportunity to discuss workload issues	3.96 ± 0.86	4.57 ± 0.68	−3.854	<.001
I have the opportunity to discuss clinical problems encountered during handover	4.09 ± 0.78	4.51 ± 0.75	−2.708	.008
I have the opportunity to ask about problems I do not understand	4.34 ± 0.70	4.60 ± 0.61	−1.800	.063
I find that handover does not take too much time	3.53 ± 0.86	4.15 ± 1.02	−3.175	.002
The information I receive during handover is all related to the patient	4.06 ± 0.60	4.47 ± 0.72	−2.953	.004
I can obtain information about the patient in a timely manner	3.98 ± 0.74	4.40 ± 0.78	−2.736	.007
Total score	52.51 ± 5.21	58.21 ± 7.24	−4.380	<.001

Data are mean ± standard deviation.

PEWS = pediatric early warning score, SBAR = situation, background, assessment, recommendation.

The total nurse handover evaluation score was also higher during the intervention period than during the control period (58.21 ± 7.24 vs 52.51 ± 5.21, *t* = −4.380, *P* < .001; Table 3). Significant improvements were observed in receiving the latest information, fully obtaining patient information, receiving important information, focusing on information, consulting colleagues during difficulties, discussing workload and clinical problems, perceived time efficiency, relevance of handover information, and timely access to patient information. Several items, including organizing received information clearly, ease of handing over information provided by colleagues, and opportunities to ask about unclear problems, showed numerical improvement but did not reach statistical significance.

### 3.3. Family bedside handover perception

The total family bedside handover perception score was higher during the intervention period than during the control period (63.23 ± 3.51 vs 55.59 ± 3.71, *t* = −21.601, *P* < .001; Table 4). Improvements were observed in most items, including helping caregivers meet care-related needs, protecting privacy, encouraging participation in nursing care, communicating about the child’s condition at bedside, improving caregiver comfort, ensuring understanding of helpful information, listening attentively, sharing important information during handover, showing respect, asking whether caregivers had questions or concerns, informing caregivers of essential examination procedures, and answering questions and concerns. However, several communication-experience items, including understandable guidance, politeness and friendliness during nursing care, perceived teamwork among nurses, information about follow-up plans, and identification of the responsible nurse, showed no statistically significant between-period differences.

**Table 4:** Family bedside handover perception scale scores in the control and observation groups.

Item	Control group (n = 206)	Observation group (n = 212)	*t*	*P*-value
1. Help me achieve my needs	3.30 ± 0.54	4.36 ± 0.64	−18.304	<.001
2. Protect privacy	3.98 ± 0.21	4.65 ± 0.58	−15.636	<.001
3. Encourage me to participate in nursing	3.00 ± 0.27	3.92 ± 1.14	−11.115	<.001
4. Bedside communication about the condition	3.01 ± 0.26	3.88 ± 1.14	−10.716	<.001
5. Make me feel comfortable	3.27 ± 1.14	3.69 ± 1.22	−3.655	<.001
6. Ensure that I understand helpful information	2.94 ± 0.58	3.98 ± 0.28	−23.498	<.001
7. Do not interrupt me and listen attentively	2.85 ± 1.12	3.09 ± 0.46	−2.945	.003
8. Share important information during handover	3.07 ± 0.81	4.00 ± 0.31	−15.581	<.001
9. Guide me in a way I can understand	3.44 ± 0.96	3.53 ± 0.99	−0.952	.341
10. Respect me	3.89 ± 0.75	4.03 ± 0.27	−2.613	.009
11. Be polite and friendly during nursing	3.83 ± 0.88	3.92 ± 0.76	−1.114	.266
12. Ask me whether I have questions or concerns	2.71 ± 0.87	3.85 ± 0.87	−13.445	<.001
13. Nurses work well together	2.95 ± 0.92	3.08 ± 1.02	−1.402	.162
14. Inform me of follow-up plans	3.24 ± 1.32	3.33 ± 0.80	−0.836	.404
15. Let me know who my responsible nurse is	3.91 ± 0.85	4.00 ± 0.82	−1.074	.283
16. Inform me of essential examination procedures	3.03 ± 0.69	3.91 ± 0.85	−11.640	<.001
17. Answer my questions and concerns	3.18 ± 0.72	3.41 ± 0.92	−2.732	.007
Total score	55.59 ± 3.71	63.23 ± 3.51	−21.601	<.001

Data are mean ± standard deviation.

### 3.4. Handover errors, efficiency, and safety-related outcomes

The handover error rate was lower during the intervention period than during the control period (27/395, 6.84% vs 69/384, 17.97%; *χ*^2^ = 22.337, *P* < .01; Table 5). Report-writing time was shorter during the intervention period than during the control period (2.11 ± 0.41 minutes vs 4.51 ± 0.32 minutes, *t* = 90.906, *P* < .001). Bedside handover time was also shorter during the intervention period (3.94 ± 0.63 minutes vs 5.97 ± 0.31 minutes, *t* = 56.809, *P* < .001; Table 5).

**Table 5:** Handover errors, efficiency indicators, unplanned PICU transfer, and nursing adverse events.

Outcome	Control group	Observation group	Test statistic	*P*-value
Handover errors, n/N (%)	69/384 (17.97)	27/395 (6.84)	*χ*^2^ = 22.337	<.01
Handover report-writing time, min	4.51 ± 0.32 (n = 384)	2.11 ± 0.41 (n = 395)	*t* = 90.906	<.001
Bedside handover time, min	5.97 ± 0.31 (n = 384)	3.94 ± 0.63 (n = 395)	*t* = 56.809	<.001
Unplanned PICU transfer, n/N (%)	8/206 (3.88)	2/212 (0.94)	*χ*^2^ = 3.868; Fisher exact *P* = .059	*χ*^2^ *P* = .049
No unplanned PICU transfer, n/N (%)	198/206 (96.12)	210/212 (99.06)		
Nursing adverse events, n/N (%)	23/206 (11.17)	10/212 (4.72)	*χ*^2^ = 5.974	.015
No nursing adverse events, n/N (%)	183/206 (88.83)	202/212 (95.28)		

Data are n/N (%) or mean ± standard deviation. PICU, pediatric intensive care unit. Fisher’s exact test was added for unplanned PICU transfer because of the small number of events; therefore, this outcome was interpreted as a lower observed numerical rate rather than a statistically robust reduction.

The unplanned PICU transfer rate was numerically lower during the intervention period than during the control period (2/212, 0.94% vs 8/206, 3.88%; *χ*^2^ = 3.868, *P* = .049). However, because of the small number of events, the comparison was borderline and not statistically significant by two-sided Fisher’s exact test (*P* = .059). Nursing adverse events were less frequent during the intervention period than during the control period (10/212, 4.72% vs 23/206, 11.17%; *χ*^2^ = 5.974, *P* = .015; Table 5). These safety-related findings were interpreted as observed between-period differences rather than as direct evidence that the intervention alone reduced these outcomes.

## 4. Discussion

### 4.1. Principal findings

This single-center nonconcurrent controlled study found that the period after implementation of a PEWS-integrated electronic SBAR handover model was associated with higher handover quality, fewer handover errors, higher nurse and family handover evaluations, shorter documentation and bedside handover time, and fewer observed nursing adverse events. The unplanned PICU transfer rate was numerically lower during the intervention period, but the small number of events and borderline Fisher exact result require particularly cautious interpretation. These findings suggest that integrating structured communication, pediatric early warning assessment, and electronic information extraction may be useful for improving pediatric handover practice. However, because the study was not randomized and the 2 comparison periods were separated in time, the results should be interpreted as associations with implementation rather than as definitive causal effects.

### 4.2. Handover quality and nurse evaluation

Pediatric handover involves large amounts of information, including rapidly changing symptoms, vital signs, medication orders, specialist assessments, potential complications, and family communication needs. Routine handover can be affected by nurses’ experience, memory, time pressure, and differences in communication style.^[19–21]^ The PEWS-integrated electronic SBAR model provided a standardized sequence and a structured checklist for transferring essential information. Compared with the routine SBAR period, the intervention period showed higher bedside handover quality scores and lower handover error rates. This may be because the electronic template reduced reliance on memory and helped nurses verify key information before bedside communication.

The improvement in nurse handover evaluation scores may also be explained by better information accessibility and clearer communication responsibilities. The model allowed automatically extracted clinical information to be reviewed and supplemented before bedside handover. It also made PEWS and other risk assessments visible within the A (Assessment) component of SBAR, thereby supporting more risk-focused nursing judgment. Similar studies have reported that structured or modified SBAR handover can improve communication quality and nurse perception of handover.^[22–26]^

### 4.3. Family perception of bedside handover

Family bedside handover perception scores were higher during the intervention period. Pediatric care depends heavily on cooperation between nurses and caregivers, and caregivers often experience anxiety when information is fragmented or unclear. The structured bedside handover process made nursing assessment, treatment priorities, and next-shift recommendations more visible to family members. This may have increased family members’ understanding of the care plan and their sense of participation. Nevertheless, satisfaction is subjective and may be influenced by factors beyond handover, such as disease severity, waiting time, staff attitude, and family expectations. Therefore, the observed improvement should be interpreted in the context of the overall implementation period.

### 4.4. Handover efficiency

The intervention period showed shorter report-writing time and bedside handover time. The electronic handover report used HIS-based data capture, nurse verification, and standardized output, which likely reduced duplicated documentation and improved the speed of information retrieval. The reduction in bedside handover time did not necessarily mean that less information was communicated; rather, information may have been communicated in a more organized and focused manner. These findings are consistent with the intended function of electronic handover tools, but they should also be interpreted with consideration of potential learning effects, because nurses became more familiar with the workflow after training and repeated use.

### 4.5. Unplanned PICU transfer and nursing adverse events

The observed rate of nursing adverse events was lower during the intervention period, and the unplanned PICU transfer rate was numerically lower. PEWS may support earlier recognition of clinical deterioration, while SBAR may help nurses communicate risks and recommended actions more clearly.^[18,27,28]^ However, these outcomes are multifactorial and may be affected by disease severity, seasonal case mix, nurse staffing, physician decision-making, PICU bed availability, and other concurrent safety practices. The number of unplanned PICU transfers was particularly small, and the comparison was borderline when Fisher’s exact test was used. Therefore, this finding should be described as a lower observed rate during the intervention period, not as proof that the model alone directly reduced PICU transfers. The adverse-event finding is more statistically stable than the PICU transfer finding, but it should still be interpreted within the limitations of the nonconcurrent design.

### 4.6. Strengths and limitations

This study has several strengths. The intervention was developed according to pediatric care characteristics, incorporated PEWS into the assessment component of SBAR, and used electronic data extraction to support standardized bedside communication. The study also assessed multiple outcomes from nurse, family, efficiency, and safety perspectives. Inter-rater agreement for handover error assessment was high, supporting the reliability of this outcome.

Several limitations should be acknowledged. First, the nonconcurrent controlled design is vulnerable to temporal and seasonal confounding. Winter and summer pediatric disease patterns and workload may differ, and not all differences could be measured. Second, the same nurses participated in both periods, so training, repeated assessment, and familiarity with the study process may have contributed to improvement. Third, the intervention was a multicomponent package, and the independent effects of SBAR restructuring, PEWS integration, electronic extraction, and staff training could not be separated. Fourth, this was a single-center study from 3 pediatric wards, which limits generalizability. Fifth, safety-related outcomes had relatively low event counts, especially unplanned PICU transfers; therefore, *χ*^2^ results for these outcomes may be unstable, and Fisher’s exact testing should be considered when interpreting statistical significance. Finally, long-term sustainability, technology-related risks, and cost-effectiveness were not evaluated.

### 4.7. Implications for practice and research

The findings suggest that a PEWS-integrated electronic SBAR handover model may provide a practical framework for improving pediatric handover standardization and efficiency. For clinical implementation, hospitals should ensure that electronic information is verified by nurses rather than accepted passively, develop contingency procedures for system failure or data errors, and provide periodic training to maintain nurses’ clinical observation and critical thinking skills. Future research should use multicenter prospective or randomized designs, extend follow-up, evaluate patient acuity and workload more comprehensively, and apply process evaluation to determine which components of the intervention contribute most to improvement.

## 5. Conclusion

In this single-center nonconcurrent controlled study, implementation of a PEWS-integrated electronic SBAR handover model was associated with higher pediatric bedside handover quality, better nurse and family handover evaluations, fewer handover errors, shorter documentation and bedside handover time, and fewer observed nursing adverse events during the intervention period. The unplanned PICU transfer rate was numerically lower, but this finding should be interpreted cautiously because the number of events was small and the Fisher exact result was borderline. Because the study was nonrandomized, conducted in different time periods, and included limited safety-related events, these findings should not be interpreted as definitive evidence of direct causal effects. The model appears promising for standardizing pediatric handover, but further multicenter prospective studies are needed to confirm its effectiveness, sustainability, and cost-effectiveness.

## Acknowledgments

The authors thank the pediatric nurses and participating families for their support of this study.

## Author contributions

**Conceptualization:** Bo Liu.

**Data curation:** Bo Liu.

**Supervision:** Bo Liu.

**Formal analysis:** Xiaowan Gu, Zaoqiong Gao, Jiangbo Ba, Qiang Fu, Ting Chen, Shengnan Zhao.

**Writing – original draft:** Huali Huang.

**Writing – review & editing:** Huali Huang.
